# Addressing the Opioid Crisis through an Interdisciplinary Task Force in Cincinnati, Ohio, USA

**DOI:** 10.3390/pharmacy8030116

**Published:** 2020-07-09

**Authors:** Neil J MacKinnon, Ellena Privitera

**Affiliations:** 1 Winkle College of Pharmacy, University of Cincinnati, Cincinnati, OH 45267, USA; 2 College of Medicine, University of Cincinnati, Cincinnati, OH 45267, USA; privitem@mail.uc.edu

**Keywords:** opioids, addiction, health policy, interdisciplinary, academic health center

## Abstract

Opioid use has been a topic of concern in recent years in the United States, causing thousands of deaths each year. Ohio is one of the states hit hardest by the epidemic, and its state and local governments have responded with comprehensive health policies. Cincinnati, located in the southwest region of Ohio, is one of the epicenters of the state’s opioid crisis. Responding to the needs of their community, the University of Cincinnati (UC) and its affiliate health system, UC Health, have brought together leaders in research, clinical practice, and education to form the UC/UC Health Opioid Task Force. By encouraging interdisciplinary partnerships, the Task Force is pioneering new ways to understand, prevent, and treat opioid use disorder, while preparing the next generation of healthcare professionals. Additionally, collaboration across departments in UC Health has improved access to treatment and recovery resources for hundreds of patients. Leading educational events, supporting local agencies, and participating in government initiatives have further solidified UC and UC Health’s role as a stakeholder in this crisis, showcasing how academic health centers are critical to promoting public health.

## 1. Overview of the Opioid Crisis in the United States and in The State of Ohio

In the late 1990s, the number of opioid prescriptions in the United States began to rise considerably as pain management became an increasing priority in patient care and pharmaceutical companies aggressively marketed the safety and utility of their opioids. By 2017, opioids caused more deaths per year than the HIV/AIDS epidemic did at its peak in the 1990s and caused more deaths than from car crashes or guns [1]. Approximately 128 people in the US died every day due to opioid overdose in 2018 [2], and the Centers for Disease Control and Prevention (CDC) estimated that prescription opioid misuse, the resulting care to treat patients who suffer from addiction, the loss of productivity, and the burden on the criminal justice system costs the US $78.5 billion annually [3]. 

There are many factors contributing to the opioid epidemic. Some have explored the relationship between the direct-to-physician marketing of opioids by pharmaceutical companies and mortality from prescription opioid overdoses. One study looked at $39.7 million in opioid marketing targeted at 67,507 physicians across 2208 counties in the US from 2013 to 2015. The authors of that study concluded that the “marketing of opioid products to physicians was associated with increased opioid prescribing and, subsequently, with elevated mortality from overdoses” [4]. 

**Figure 1:**
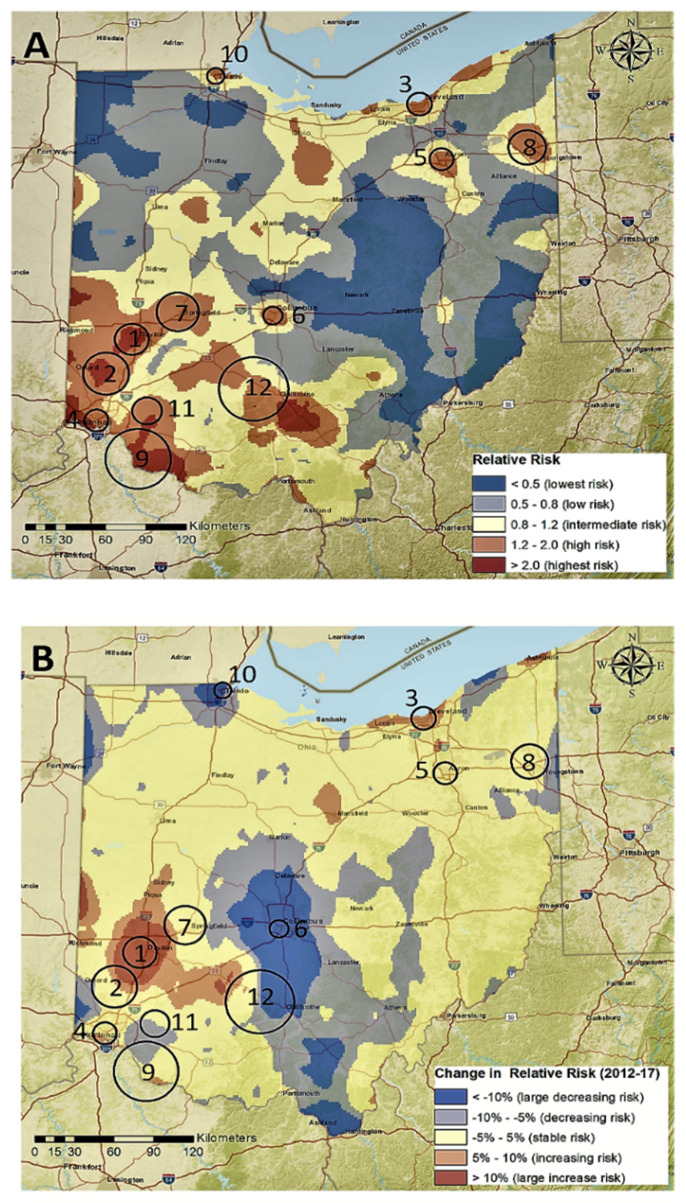
(**A**) Spatial distribution of the relative risk of death by prescription opioid overdose in Ohio (2010–2017), and (**B**) change in the relative risk between 2010 to 2017.

The State of Ohio is among the states that have felt the greatest burden of this epidemic. For example, Ohio is one of eight states whose opioid mortality rate doubled every three years from 1999 to 2016, and Ohio has the fifth-highest rate of overdose deaths in the US [1]. Unintentional drug overdoses have become Ohio’s leading cause of injury-related death. A review of the Ohio Department of Health data from 2010–2017 shows that prescription opioid overdose risk is clustered throughout the state, with most hotspots in near cities in Southwest Ohio, with additional hotspots near large cities in Northern Ohio (Figure 1) [5]. The highest prescription opioid overdose mortality rates were found in the White male population (ages 30–39), while Black males experienced the fastest estimated annual increase of the opioid overdose mortality rate in the same time period [5].

In response to the opioid epidemic, the Trump Administration released the National Drug Control Strategy to focus the federal government on prevention, reducing barriers to treatment, and reducing the availability of illicit drugs. The National Institutes of Health (NIH) is investing in hundreds of research projects nationwide through its Helping to End Addiction Long-term (HEAL) initiative. Several states, including Ohio, are suing opioid manufacturers for their role in the epidemic. Ohio’s former governor, John Kasich, developed one of the nation’s most aggressive and comprehensive approaches to combatting opioid use in 2011 with the Cabinet Opiate Action Team. The team invested over $1 billion USD per year to promote responsible opioid use, reduce supply, prevent overdose, and expand access to naloxone [6]. Compared to 2011, 81 million fewer doses of opioids were dispensed in 2015, and the proportion of unintentional drug overdose deaths involving prescription opioids reduced from 45% to 22% [7]. However, the rate of unintentional overdose deaths in Ohio continued to rise, alongside increasing uses of synthetic opioids. In 2015, 40% of overdose deaths involved fentanyl, and, in 2018, fentanyl was involved in nearly 73% of overdose deaths [8]. Ohio’s current governor, Mike DeWine, launched the RecoveryOhio initiative in 2019 to further address the epidemic by expanding treatment and recovery services, promoting prevention and harm reduction, addressing stigmas associated with opioid use, and improving data coordination and sharing. 

One research team at the University of Cincinnati has actively evaluated a number of health policies and strategies aimed at addressing the opioid epidemic in that state. In a cross-sectional, statewide survey of emergency department medical directors in 2016, the team evaluated the implementation of prescribing guideline issues by the Ohio Department of Health to address the prescribing of opioids and other controlled substances in emergency department and acute care facilities. The survey received a 92% response rate, and the majority of the respondents (71%) believed that the guidelines did help to reduce inappropriate opioid prescribing [9]. In a related qualitative study consisting of interviews with emergency physicians in Ohio, concerns were expressed with the guidelines, including a perceived lack of organizational responsibility on behalf of hospitals to address inappropriate opioid prescribing [10]. These physicians also commented on prescribers’ unconscious bias toward patients at high risk of opioid overdose and prescribers’ lack of education and awareness about take-home naloxone [11]. The State of Ohio’s Prescription Drug Monitoring Program (PDMP) was implemented in April 2015, and an interrupted time series analysis was conducted to evaluate the effectiveness of this program in reducing opioid and benzodiazepine dispensing. The analysis concluded that there was a statistically significant decrease in the monthly quantity of these prescriptions dispensed in Ohio following implementation [12]. In a separate, interrupted time series analysis, the effectiveness of an Ohio law allowing pharmacists to dispense naloxone without a prescription was evaluated. After implementation of this law in July 2015, the amount of naloxone dispensed increased by 2328%, thus greatly enhancing access to this drug [13].

## 2. The University of Cincinnati/UC Health Opioid Task Force 

Hamilton County, where the University of Cincinnati (UC) and UC Health are located in Southwest Ohio, has among the highest opioid overdose and mortality rates in the state. Between 2009–2018, Hamilton County averaged 40.5 deaths per 100,000 people, compared to the state average of 30.5, highlighting the need for effective interventions and health professionals equipped to assist Southwest Ohioans [8]. Community groups such as the Hamilton County Addiction Response Coalition (HCARC) have responded by providing prevention education, improving treatment availability, and facilitating harm reduction programs. Additionally, police officers and first responders in the Greater Cincinnati region have formed quick response teams (QRTs), which visit people’s homes following overdose events to provide a referral for addiction treatment and social services. QRTs have served as a model for public safety officers to further address the opioid epidemic, having spread across Ohio and to neighboring states. 

As one of Ohio’s leading public research universities, the UC and its affiliate healthcare system, UC Health, aim to work alongside government and community leaders to address the opioid epidemic. Together, UC/UC Health make up the Greater Cincinnati region’s only academic health center, simultaneously delivering quality healthcare, performing research, and training future health professionals. Furthermore, UC Health plays a prominent role in the Greater Cincinnati region’s health and safety, with over 40 locations, including the UC Medical Center (UCMC). UCMC is the region’s only level 1 trauma center, capable of providing total care for all aspects of injuries.

Central to the success of UC/UC Health’s efforts to combat Ohio’s opioid epidemic is the Opioid Task Force. Cochaired by Melissa DelBello, MD, Dr. Stanley and Mickey Kaplan, Professor and Chair of Psychiatry at the UC College of Medicine (CoM), and Neil J MacKinnon, PhD, Dean and Professor of the James L Winkle College of Pharmacy (JLWCoP), this group serves as the coordinating body for the opioid-related activities performed across the university and UC Health. The Opioid Task Force was created in 2017 to bring together researchers, educators, clinicians, and leaders/advocates who are dedicated to having a positive impact on opioid addiction locally, regionally, and nationally. Since its inception with 40 members in 2017, the task force has grown in the size and scope of its membership, including 70 members as of 2020, representing almost every college at UC, alongside a range of departments in UC Health. To achieve its goals, the task force is divided into four interdisciplinary working groups: research, education, clinical practice, and community outreach. In carrying out these complementary activities, UC and UC Health exemplify the critical role academic health centers play in effectively addressing a widespread public health emergency.

Through cutting-edge research funded by federal agencies, including the National Institutes of Health (NIH) and the US Department of Federal Affairs, UC/UC Health are pioneering new treatment and prevention methods. Research is focused on patient-centered intervention methods, posttreatment behavioral changes, postpartum treatment retention, teleconference education, overdose education, and naloxone distribution. In 2019, the Substance Abuse and Mental Health Services Administration (SAMHSA) provided UC with $15.1 million over four years to implement the Project HEALing Communities Study. UC faculty are working in collaboration with RecoveryOhio, the Ohio State University, Case Western Reserve University, and several other universities and community organizations to implement and evaluate intervention approaches in 19 counties across Ohio.

To prepare Ohio’s future workforce to address this epidemic, the task force has integrated addiction education into coursework in the College of Nursing (CoN), College of Allied Health Sciences (CAHS), CoM, and the JLWCoP to ensure that all health professional students are equipped to address all aspects of opioid addiction. Using a $1 million grant from the SAMHSA, the CAHS has implemented an interprofessional Screening, Brief Intervention, and Referral to Treatment training course to students in CoN, CoM, JLWCoP, and CAHS, with hopes to expand this program to include law and criminal justice students in the future. Faculty from CAHS, CoN, and UC’s Fire Science program have collaborated with local fire departments and Cincinnati’s Center for Addiction Treatment to teach students how to link people with substance use disorders to social and healthcare services.

Additionally, UC Health provides wide-ranging clinical care for more than 600 patients suffering opioid use disorder (OUD), tailored to the specific needs of diverse patient populations. For example, the Perinatal Addictions Clinic is a collaborative effort between several different disciplines that has provided perinatal, addiction, and mental health treatments to women with OUD, serving over 125 mother-infant dyads since 2016. A recent pilot program for a medication-assisted treatment (MAT) pathway within the Center for Emergency Care at UC Medical Center provides screening, medications for OUD, addiction counseling, and ensures early/next-day entrance into recovery programs at UC Health and other community resources. The program has trained over 100 emergency medicine care providers in MAT, waivered 35 providers to provide MAT in the ED, and has treated 56 patients with MAT in the emergency department since early 2019.

**Figure 2:**
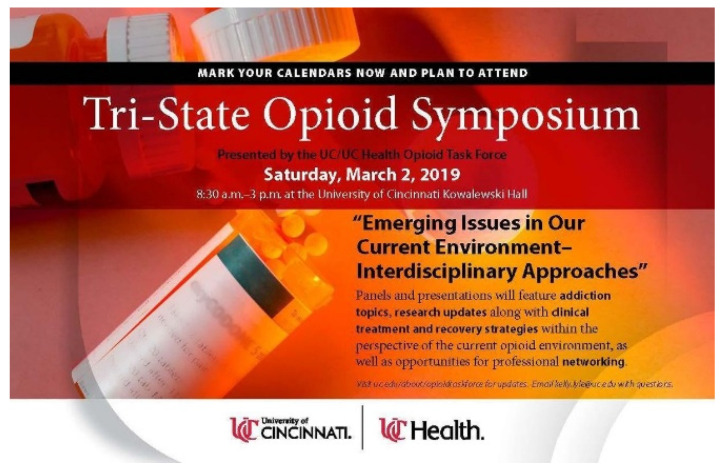
Flyer for the 2019 Tri-State Opioid Symposium.

To improve public understanding of the opioid epidemic and its impact, the task force has published articles and hosted two tri-state opioid symposiums, inviting scientists and leaders from around the country to inspire and engage participants. Figure 2 contains the flyer for the 2019 Tri-State Opioid Symposium, hosted by the Task Force. Additionally, service-learning initiatives led by UC’s College of Arts & Sciences engage undergraduate students in addressing the social implications of OUD as they provide job-readiness training at Cincinnati’s Center for Addiction Treatment. Finally, task force members have played integral roles in the activities of local agencies and groups, including the HCARC, Cincinnati Exchange Project, and RecoveryOhio. 

Future goals for the task force’s efforts are outlined in Appendix A. The onset of the COVID-19 epidemic has challenged UC Health and other providers to change their patient care protocols, forced educators to adapt to remote learning, and has revised research projects. At the same time, the rise of synthetic opioid use and the use of other illicit drugs (including methamphetamine, cocaine, and benzodiazepines) presents new challenges for experts attempting to address Ohio’s addiction epidemic. The task force hopes to improve the understanding of the opioid epidemic by continuing to foster interdisciplinary collaboration, such as the partnership between geographers and pharmacists that resulted in the geospatial map of Ohio’s opioid epidemic (Figure 1). The size and breadth of the task force can serve both as a strength and challenge, as the leadership team must balance mainly virtual communications between a large group of professionals. Regular in-person meetings of the task force as a whole offer new opportunities for collaboration and creativity, while the committee structure creates a sense of direction for the group.

Additionally, funding for the task force comes largely from federal grants for individual projects. To establish further financial and institutional support for interdisciplinary research, the task force leadership aims to establish a new center on addiction within the university. Finally, the task force aims to expand interprofessional education opportunities to students in different disciplines and to continue supporting clinicians developing innovative treatment pathways for patients suffering OUD.

## 3. Conclusions

The UC/UC Health Opioid Task Force is coordinating a campus-wide response to the opioid epidemic, which has especially affected the Cincinnati community. Bringing together a wide range of clinicians, researchers, educators, and community leaders has allowed for the academic health center to leverage its wide range of experts, forming unique partnerships that have enhanced our understanding and ability to approach this complex crisis. The task force has become a key player in promoting the Cincinnati region’s interests by connecting with state and nationwide initiatives to combat the epidemic, exemplifying how an academic health center can use collaboration to simultaneously address the needs of and advocate on behalf of their communities.

There are some key lessons learned and limitations from the experience of the UC/UC Health Opioid Task Force that could be applied to other groups hoping to address the opioid crisis by similar means. First, because efforts of the task force are so diffuse and broad, the exact impact of the task force is difficult to ascertain. There has been a significant decrease in deaths from unintentional drug overdoses during the period of operation of the task force—for example, a drop from 4854 deaths in 2017 to 3764 deaths in Ohio [8]—but the role of the task force in helping to decrease these deaths cannot be assessed. Secondly, the task force was intentionally established to be interdisciplinary, but it does make it more challenging to assess the impact of discipline-specific activities. For example, the pharmacy department at UC Health has been an essential part of clinical practice changes at UC Health related to the continuity of care for patients with addiction, but our interventions cannot parse out the role of pharmacists on patient outcomes. Finally, as presented in the appendix, it is expected that the nature of the task force itself will evolve and change, most likely into a Center for Drug Addiction.

## Appendix A

**UC/UC Health Opioid Task Force Goals (Updated Winter 2020)**

Over the next three years, the UC/UC Health Opioid Task Force aims to achieve the following goals and to establish an addiction center within the University of Cincinnati. Other institutions in Ohio are developing addiction centers. Ideally, a University of Cincinnati Addiction Center would include research, education, and clinical services. The Task Force itself may eventually transition to an advisory board to the center once it is established.

**Goal** **1**:
*Train Students across Disciplines to be Leaders in Substance Use Prevention, Screening, and Treatment.*

1. Sustain and expand the motivational interviewing and screening, brief intervention, and referral to treatment (SBIRT) course so that all students can receive SBIRT training, including students in the College of Law and the School of Criminal Justice.
  - This would require the hire of SBIRT faculty, a course director, and an administrative assistant to coordinate schedules, logistics, and be present at all trainings.
  - This would need to explore future partnerships with additional schools and with jails and corrections officers.
  - Current outcomes: A review of the course showed that 98% of students strongly agreed or agreed with the statement, “The training enhanced my skills in this topic area” at baseline and 98.7% at a 30–day follow-up. Ninety-six percent of students strongly agreed or agreed with, “The material presented in this class will be useful to me in dealing with substance abuse” and 98% at a 30–day follow-up.
2. Incorporate integrative health into treatment and training via partnership with the Center of Integrative Health and Wellness.
3. Ensure that every health professional student, at a minimum, has the cultural and clinical competencies needed to work with patients with substance use disorders. This would include an expansion of the collaborative health care team course that began with medical and pharmacy students in 2019.

**Goal** **2**:
*Establish The University of Cincinnati as a Top University for Addiction/Substance Abuse Research.*

Within the addiction center, a research arm would be established that would focus on career development, interdisciplinary collaboration, the generation of preliminary data, the acquisition of instrumentation, and other infrastructure supports and moderate-sized initial and interim grant supports for new programs of research. This would:

1. establish addiction-related research as one of the highest priorities among leadership at UC and UC Health.
2. retain UC researchers and accelerate their ability to garner extramural research funding by providing protected time, space, and seed grants for pilot projects.
3. provide funded support to establish multidisciplinary, multi-departmental, and interprofessional collaborations and to support junior faculty research.
  - The expectation would be that investigators supported by the center in the first five years would independently generate a combined $5 million of new research funding by year six (i.e., progress towards sustainability).

This initiative would ask UC/UC Health to provide financial support for equipment and grants for faculty doing addiction research, in addition to the hiring of an administrative assistant/grants manager, an administrative principal investigator (PI), PI for the interdisciplinary collaboration, and a PI for career/research development.
**Goal** **3**: Identify and Provide Resources for Healthcare Leaders to Implement Clinical Programs within Our Health and Education System.

1. Implement medication-assisted treatment (MAT) pathways within the West Chester Hospital Emergency Department and within the UCMC Emergency Department Early Intervention Program (EIP) to ensure the identification, assessment, and linkage to continuing outpatient care within 24 hours from the initial emergency department (ED) visit.
  - Current outcomes: 106 emergency medicine care providers have been educated in ED MAT, 44 emergency medical (EM) care providers have become Data 2000 waivered, and 170 patients have been treated with MAT in the ED in 2019 and referred to treatment with an appointment within 24 hours of the ED visit.
2. Get a comprehensive care team for people on opiate medicine for chronic pain and want to get off it.
3. Achieve maximal impact with the addiction services currently in place by:
  - resolving billing and organizational issues in recently expanded addiction services (for example, Medicaid billing for methadone, and the integration of hospital-based and outpatient resources).
  - investing in three providers to provide case management for addiction patients.
  - increasing consult availability by exploring a hospitalist addiction model, as well as a dedicated social worker.
  - promoting services that we have in place to the community.
4. Implement screening into clinical practice across UC Health practice locations. This includes current initiatives to screen pregnant women.
5. Reduce deaths due to opioid use by increasing the number of physicians waivered to initiate medications for opioid use disorder (MOUD) in the ED and continuing to engage the infectious disease services in the task force.
